# Cardiac MRI in patients with Fontan circulation: assessing risk factors for adverse outcomes

**DOI:** 10.1136/openhrt-2025-003306

**Published:** 2025-05-21

**Authors:** Sabina Ericsson, Riitta Paakkanen, Marko Taipale, Emmi Helle, Juha Peltonen, Alma Kormi, Teemu Vepsäläinen, Ilkka Mattila, Tommi Pätilä, Laura Martelius, Tiina Ojala

**Affiliations:** 1 New Children’s Hospital Pediatric Research Center, Helsinki University Hospital, Helsinki, Finland; 2 Stem Cells and Metabolism Research Program, Faculty of Medicine, University of Helsinki, Helsinki, Finland; 3 Population Health Unit, Finnish Institute for Health and Welfare, Helsinki, Finland; 4 HUS Diagnostic Center, Radiology, Helsinki University Hospital and University of Helsinki, Helsinki, Finland; 5 Department of Cardiac and Transplantation Surgery, New Children’s Hospital, Helsinki University Hospital and University of Helsinki, Helsinki, Finland

**Keywords:** Magnetic Resonance Imaging, Fontan Procedure, Heart Defects, Congenital

## Abstract

**Background:**

Cardiac magnetic resonance (CMR) imaging provides critical insight into the prognosis of Fontan patients, enhancing our understanding of their long-term outcomes. This study aimed to investigate the prognostic role of CMR in a carefully selected cohort of Fontan patients with the highest initial likelihood of survival.

**Methods:**

This retrospective nationwide cohort study included 148 Fontan patients who underwent post-Fontan CMR imaging in Finland between 2017 and 2023. The primary endpoint was death or listing for heart transplant. The secondary endpoint was myocardial fibrosis determined by native T1 mapping measured by CMR.

**Results:**

The median time from the Fontan procedure to CMR examination was 10.8 years, with a median post-CMR follow-up of 2.55 years. Six patients (4.1%) reached the primary endpoint. Significant haemodynamic risk factors for the primary endpoint included worse global longitudinal strain (p=0.03), worse global circumferential strain (p<0.001) and reduced ejection fraction (p=0.04). Notably, patients with decreased myocardial function showed higher native T1-mapping values. Additional clinical risk factors that were associated with the primary endpoint included arrhythmias (p=0.01), protein-losing enteropathy (p=0.01), New York Heart Association functional class ≥2 (p<0.001) and liver cirrhosis (p=0.01).

**Conclusions:**

CMR provides critical insights into long-term outcomes in Fontan patients. In our prioritised cohort, characterised by an initially high likelihood of survival, the observed risks of adverse outcomes corroborate findings from higher mortality cohorts. This underscores the importance of myocardial function and native myocardial T1 mapping in risk assessment, reaffirming CMR’s role in effective risk stratification for this population.

**WHAT IS ALREADY KNOWN ON THIS TOPIC**

- Integration of cardiac magnetic resonance (CMR) data with clinical parameters, such as ventricular dysfunction, systemic right ventricle dominance and protein-losing enteropathy, is crucial for risk stratification in individuals with univentricular hearts. Myocardial strain and native T1 mapping show potential as early markers of impaired ventricular function and adverse outcomes. While these advancements enhance early risk detection using CMR, most studies are largely carried out with heterogeneous cohorts and studies on prioritised patient cohorts with high likelihood of survival remain limited.

**WHAT THIS STUDY ADDS**

- This study confirms the prognostic value of CMR-derived strain measurements (global longitudinal strain and global circumferential strain) and myocardial fibrosis (native T1 mapping) in a rigorously selected cohort of Fontan patients, highlighting their associations with adverse outcomes.

**HOW THIS STUDY MIGHT AFFECT RESEARCH, PRACTICE OR POLICY**

- These findings help to identify patients at increased risk for adverse outcomes, allowing for more precise risk stratification in the early stages. They influence clinical practice by optimising treatment plans, improving resource allocation and promoting the routine use of these imaging biomarkers in follow-up protocols. Additionally, they inform policy by advocating for standardised CMR-based surveillance strategies for Fontan patients.

## Introduction

 The Fontan procedure represents a significant milestone in the palliative care of patients afflicted with various forms of univentricular heart (UVH) defects.^1^ While it has markedly enhanced the short-term prognosis of individuals previously considered incompatible with survival, challenges are faced during follow-up.^2^ Integration of cardiac magnetic resonance (CMR) data with clinical parameters has proven instrumental in risk stratification in individuals with UVH.^3^

Key clinical risk factors for mortality and heart transplantation, such as ventricular dysfunction, systemic right ventricle (RV) dominance and protein-losing enteropathy (PLE), are well documented.^3^,^5^ Emerging evidence highlights myocardial strain and native T1 mapping as potential early markers of impaired ventricular function and adverse outcomes.^6^ These parameters could significantly improve the ability to identify risks even before clinical symptoms, such as PLE, manifest.^7^

In Finland, the selection criteria for the Fontan procedure are notably rigorous, prioritising patients with the highest likelihood of survival.^3 8^ We sought to reassess the associations among the known risk factors and adverse outcomes in our carefully selected cohort. Alongside the clinical parameters, we evaluated haemodynamic risk factors for adverse outcomes using CMR imaging. Additionally, we examined myocardial fibrosis as a secondary endpoint with native T1 mapping, given its well-established link to poor prognosis.^9^,^11^ We hypothesised that these clinical and haemodynamic risk factors may predict adverse outcomes in this carefully selected cohort, similarly to previous studies with more heterogeneous cohorts.

## Methods

### Patients

A national cohort of UVH patients in Finland who underwent post-Fontan CMR imaging between 2017 and 2023 (n=148) was retrospectively reviewed. Our centre is an academic tertiary care children’s hospital and the national referral centre for congenital cardiac surgery. The study comprised a relatively homogeneous patient cohort, in which the lower baseline risk of mortality and transplantation was a direct consequence of our stringent surgical selection criteria. Contraindications include intact atrial septum, moderate to severe tricuspid valve regurgitation and/or poor preoperative myocardial function, total or partial anomalous pulmonary vein drainage and major extracardiac malformation.^8^

The study was approved by the Research Ethics Committees of Helsinki, and it was carried out according to the Declaration of Helsinki. Patients and/or the public were not involved in the design, conduct, reporting or dissemination plans of this research.

### Clinical parameters

The UVH diagnoses included in the study were hypoplastic left heart syndrome (HLHS), double inlet left ventricle (LV), double outlet RV, unbalanced atrioventricular (AV) septal defect and right ventricular hypoplasia. Clinical data were retrieved from patient records, including type of heart defect, age at Fontan operation, time from Fontan operation to CMR, age at CMR, surgical technique used for the Norwood procedure, presence of AV valve regurgitation (AVVR), arrhythmias, New York Heart Association (NYHA) functional class and history of PLE.

Type of heart defect was classified as HLHS or other UVH. NYHA functional class was defined as II or higher. PLE was identified by hypoalbuminaemia (<30 mg/L) with abnormal alpha 1-antitrypsin faecal clearance, ascites, pleural effusion and/or diarrhoea. Liver MRI findings of cirrhosis (nodular hepatic contour and changes in volume distribution) were recorded.

### CMR analysis

CMR was performed with a 1.5 Tesla scanner (Philips Achieva or Ingenia), using gadoteric acid (279.3 mg/mL, 0.2 mL/kg). Analysis was carried out using Medis Suite including QStrain analysis software (V.4.0.70.4, Medis Medical Imaging Systems, Leiden, Netherlands). The most recent CMR examination for each patient was selected.

Imaging protocol followed the recommendations issued by the Society for CMR expert consensus group.^12^ A summary of magnetic resonance sequence parameters used at our institution has previously been published.^13^ The core of the protocol involves the assessment of branch pulmonary artery size and flow distribution, cardiac index and ventricle size and function. Ventricular volumes and function were measured at end-diastole and end-systole by tracing the endocardial and epicardial borders. The volumetric parameters were indexed for body surface area. AVVR was categorised based on severity as either mild (<30%) or significant (>30%). Vessel cross-sectional dimensions were measured from 3-D balanced steady-state free precession images acquired during diastole. Coronal fat-saturated single-shot T2-weighted non-contrast lymphangiography was used to assess neck lymphatic collaterals, classified per Biko *et al*.^14^ Aortopulmonary collateral flow was calculated from the difference in pulmonary venous and arterial flow, expressed as a percentage.

The systemic chamber was defined as the primary or largest chamber contributing to systemic circulation, with the systemic ventricle classified as LV, RV or unclassified.

**Figure 1:**
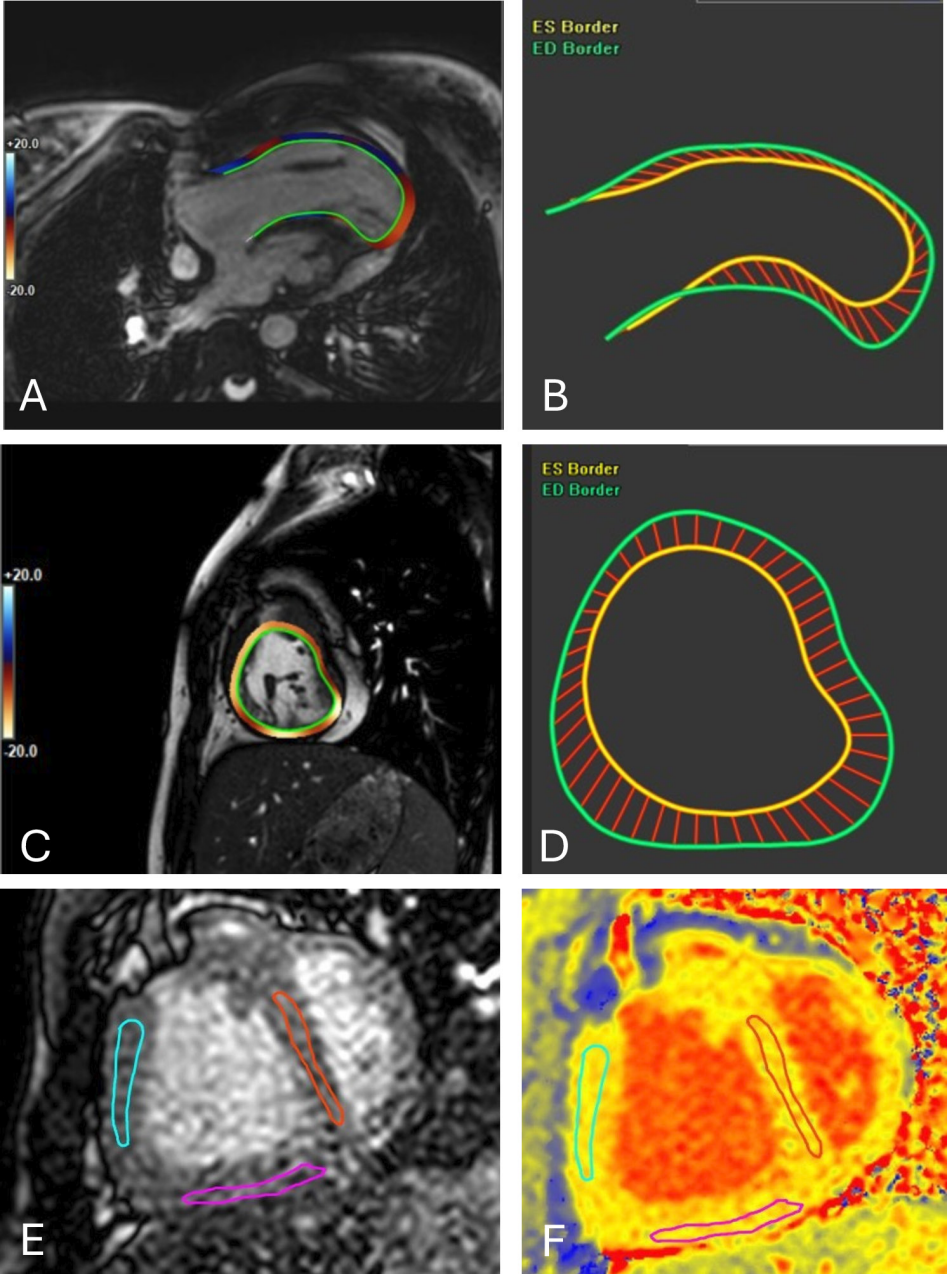
Images from GLS, GCS and native T1-mapping analysis. CMR images of (A) and (B) longitudinal and (C) and (D) circumferential strain analysis. Endocardial borders in images A and C are marked in green. Images E and F demonstrate native T1 mapping and ROIs. CMR, cardiac magnetic resonance; GCS, global circumferential strain; GLS, global longitudinal strain; ROIs, region of interests.

Global longitudinal strain (GLS) analysis was conducted using the four-chamber view of the dominant ventricle, excluding the hypoplastic ventricle. Markers were placed at the AV valve annulus on both the lateral and septal sides, and the apex at end-diastole and end-systole. Automatic tracing was manually corrected if needed. Global circumferential strain (GCS) was measured using short-axis cine images at the basal, mid and apical planes in end-diastole and end-systole. The endocardium was manually traced (figure 1). GLS and GCS are reported as negative values, with more negative values signifying better strain and more positive values signifying worse strain. Images of insufficient quality were excluded.

Native T1 mapping was acquired in three short-axis planes (base, mid and apical) by using a shortened modified Look-Locker inversion recovery sequence in the systolic heart phase (figure 1). The slice thickness was 8 mm, with a typical acquisition pixel size of 1.2×1.2 mm. Medis Suite AutoQ was used for motion correction in all analyses. T1 values were manually traced from one plane and from three different anatomical locations: septum, inferior wall and free wall. Anterior wall was omitted from the analyses due to suboptimal image quality. The tracings were made only from areas with compacted myocardium, affirmed by 2-D cine loops of the corresponding areas. In addition, severe wall motion abnormalities or areas with thinning were excluded from the analyses as these may represent surgical sequelae or other local processes rather than global myocardial changes. To compare myocardial native T1-mapping values between UVH patients and healthy individuals, we analysed mapping data from 35 healthy children (median age 10.8 years (IQR 8.0–14.8 years)) evaluated at our centre as a part of the normative CMR data collection.

### Endpoints

The primary endpoint was death or listing for a heart transplant. Follow-up time in the survival analysis was defined as the time from CMR to the endpoint or last known follow-up date. The secondary endpoint included myocardial fibrosis measured by native myocardial T1 mapping. The post-CMR clinical follow-up period had a median duration of 2.55 years (IQR 1.03–4.1 years).

### Statistical analysis

Statistical analysis was performed using IBM SPSS Statistics 29 for Windows (IBM Corp., Armonk, NY, USA). Categorical variables were analysed using χ^2^ test or Fisher’s exact test, when appropriate. Continuous values are reported as means and SD or median and IQR. For the endpoint analysis, univariate analysis was performed using an independent sample t-test or Mann–Whitney test, depending on normality. Comparisons of three groups were performed with a one-way analysis of variance. Due to the small number of patients reaching the endpoint (n=6), we refrained from conducting a multivariable analysis of the primary endpoint. Correlation between continuous variables was analysed using Spearman’s or Pearson’s correlation. Clinically significant risk factors with p values of<0.10 in the univariate analyses were included in the multivariable linear model, performed using backward stepwise method. The p values of<0.05 were considered statistically significant.

Two separate multivariable analyses were conducted for GCS association to decrease the risk of overfitting; one with clinical parameters and one with CMR-related parameters. GLS and ejection fraction (EF) were excluded from the multivariable analysis, as they measure similar functions to GCS. End-systolic volume (ESV) and end-diastolic volume (EDV) were both statistically significant in the univariate analysis, but due to their strong correlation, EDV was chosen for the multivariable analysis.

A receiver operating characteristic (ROC) curve was generated for cut-off value analysis. Area under the curve, specificity and sensitivity were calculated based on the ROC curve. Kaplan–Meier survival analysis was performed for visualisation purposes. Cases were censored at the end of the follow-up period or at the time of the endpoint.

During the study period, native T1 mapping for myocardial fibrosis assessment was introduced as a new parameter at our institution, leading to data collected for only 83 out of 148 patients. Notably, only two patients who reached the primary endpoint underwent native T1-mapping evaluation. Consequently, we performed a separate analysis of risk factors, treating myocardial T1 values as a secondary endpoint factor.

## Results

The age at the time of Fontan procedure was 3.2 years (median, IQR 2.8–3.6 years), the age at the most recent CMR examination was 14.2 (median, IQR 10.2–16.3) and the interval from the Fontan procedure to the CMR was 10.8 years (median, IQR 6.9–13.4 years). The time from the CMR to the end of follow-up was 2.55 years (median, IQR 1.03–4.1).

### Primary endpoint: mortality or heart transplant

During the follow-up period, 6 out of 148 patients (4.1%) reached the primary endpoint, which comprised two deaths and four listings for heart transplants. Three patients were transplanted, and one patient remained listed for transplantation at the end of the follow-up. A summary of patient characteristics and clinical parameters is presented in table 1.

**Table 1:** Demographic and clinical characteristics

Variable	All patients (N=148) N(%)	Transplant-free survival (N=142) N(%)	Death or listing for heart transplant (N=6) N(%)	P value
Sex (male)	85 (57)	81 (57)	4 (67)	1.00
Type of defect (HLHS)	70 (47)	67 (47)	3 (50)	1.00
NYHA functional class ≥2	14 (9)	10 (7)	4 (67)	<0.001
Arrhythmias	14 (9)	11 (8)	3 (50)	0.01
MRI-based liver cirrhosis	14 (9)	11 (8)	3 (50)	0.01
Lymph ≥ 2 (thorax)	97 (74)	95 (74)	2 (33)	0.29
Lymph (abdomen)	19 (13)	19 (13)	0 (0)	1.00
PLE	5 (3)	3 (2)	2 (33)	0.01
Shunt type				0.40
RV-PA	37 (11)	34 (24)	3 (50)	
BT	49 (33)	48 (34)	1 (17)	
PA banding	26 (18)	25 (18)	1 (17)	
AVVR (>30%)	107 (72)	102 (72)	5 (83)	1.00
Age at Fontan (years)	3.2 (2.8, 3.6)	3.2 (2.8, 3.6)	3.1 (2.5, 4.4)	0.89
Age at CMR (years)	14.2 (10.2, 16.3)	14.1 (10.3, 16.4)	15.1 (8.1, 16.7)	0.97
Time from Fontan to CMR (years)	10.8 (6.9, 13.4)	10.8 (6.9, 13.3)	11.3 (4.2, 14.1)	0.98
GLS (%)	−21.7 (5.1)	−22.0 (5.1)	−17.1 (2.5)	0.03
GCS (%)	−24.3 (5.6)	−24.7 (5.3)	−16.7 (4.0)	<0.001
EF (%)	56 (9)	57 (9)	49 (9)	0.04
Saturation at CMR (%)	97 (95, 99)	97 (95, 99)	95 (94.5, 96)	0.08
T1-mapping value (ms)	1011 (31.7)	1011 (31.7)	1031 (32.5)	0.38
CI	2.9 (0.7)	3.0 (0.7)	2.6 (0.7)	0.29
EDV (mL/m²)	85 (24)	85 (23)	95 (39)	0.29
ESV(mL/m²)	39 (20)	38 (19)	52 (30)	0.099
Pulmonary perfusion ratio	15 (14)	16 (14)	7 (7)	0.14
Aortopulmonary collaterals (%)	16 (9)	16 (9)	18 (13)	0.52

Values are reported as N (%), median (25th and 75th percentile) or mean (SD).

AVVR, atrioventricular valve regurgitation; BT, Blalock–Taussig; CI, cardiac index; CMR, cardiac magnetic resonance; EDV, end-diastolic volume; EF, ejection fraction; ESV, end-systolic volume; GCS, global circumferential strain; GLS, global longitudinal strain; HLHS, hypoplastic left heart syndrome; NYHA, New York Heart Association; PA, pulmonary artery; PLE, protein-losing enteropathy; RV-PA, right ventricle-pulmonary artery.

The CMR haemodynamic risk factors associated with the primary endpoint in the univariate analysis included worse GLS (p=0.03), worse GCS (p<0.001) and reduced EF (p=0.04). Additionally, several clinical risk factors were identified, including arrhythmias (p=0.01), PLE (p=0.01), NYHA functional class ≥2 (p<0.001) and liver cirrhosis (p=0.01), all of which significantly increased the likelihood of reaching the primary endpoint.

### CMR-based haemodynamic risk factors (GLS, GCS and EF)

We further investigated the relationships among GLS, GCS and EF with other variables through univariate and multivariable analyses. The results of the univariate analysis are detailed in online supplemental table 1. In multivariable analysis, worse GLS was significantly associated with AV valve regurgitation (b=2.4 (95% CI 0.4 to 4.5), p=0.02) and the type of defect (HLHS) (b=1.8 (95% CI 0.2 to 3.6), p=0.03) (table 2). Similarly, worse GCS was linked to NYHA functional class≥2 (b=5.7 (95% CI 2.3 to 9.0), p=0.001), type of defect (HLHS, (b=2.5 (95% CI 0.7 to 4.3), p=0.007)), liver cirrhosis (b=3.9 (95% CI 0.95 to 6.87), p=0.01) and elevated EDV (b=0.09 (95% CI 0.05 to 0.12), p<0.001) (table 2). Lower EF was associated with higher EDV values (b=0.2 (95% CI 0.13 to 0.26), p<0.001) (table 2).

**Table 2:** Multivariable analysis of CMR markers

Variable	Multivariable analysis of GLS		Multivariable analysis of GCS		Multivariate analysis of EF		Multivariable analysis of T1 mapping	
	Beta (95% CI)	P value	Beta (95% CI)	P value	Beta (95% CI)	P value	Beta (95% CI)	P value
Type of defect (HLHS)	1.8 (0.2 to 3.6)	0.03	2.5 (0.7 to 4.3)	0.01				
AVVR (>30%)	2.4 (0.4 to 4.5)	0.02						
EDV			0.09 (0.05 to 0.12)	<0.001	0.2 (0.13 to 0.26)	<0.001		
ESV							0.5 (0.3 to 0.9)	<0.001
NYHA			5.7 (2.3 to 9.0)	0.001				
Liver cirrhosis			3.9 (0.95 to 6.87)	0.01				

AVVR, atrioventricular valve regurgitation; CMR, cardiac magnetic resonance; EDV, end-diastolic volume; EF, ejection fraction; ESV, end-systolic volume; GCS, global circumferential strain; GLS, global longitudinal strain; HLHS, hypoplastic left heart syndrome; NYHA, New York Heart Association.

**Figure 2:**
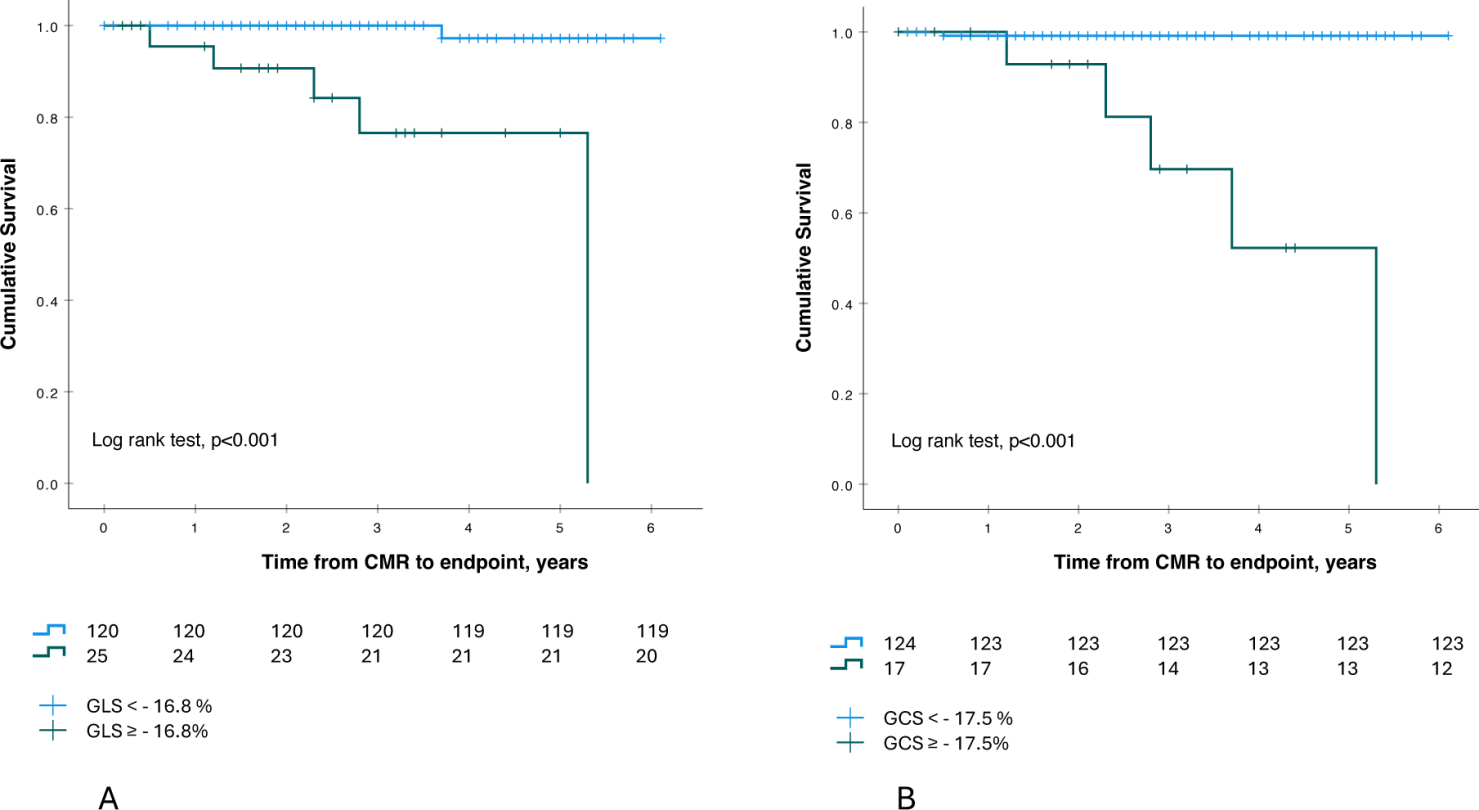
Kaplan–Meier survival curve stratified by (A) GLS and (B) GCS. Patients are stratified by higher/equal or lower (A) GLS than −16.8% and (B) GCS than −17.5%. GCS, global circumferential strain; GLS, global longitudinal strain.

We examined the critical cut-off values for GCS, GLS and EF as risk factors for the primary endpoint. ROC-curve analysis revealed that the cut-off value for GLS associated with the primary endpoint was −16.8% (sensitivity 20.0% and specificity 99.2%), with a positive predictive value (PPV) of 50.6% and a negative predictive value (NPV) of 96.7%. The cut-off value for GCS was −17.5% (sensitivity 29.4% and specificity 99.2%), yielding a PPV of 60.9% and an NPV of 99.2%. Kaplan–Meier survival curves comparing patients with GLS ≥−16.8% versus<−16.8% and GCS ≥−17.5% versus<−17.5% are presented in figure 2A,B. The cut-off value for EF related to the primary endpoint was 46.5% (sensitivity 50% and specificity 87.9%), with a PPV of 15.4% and an NPV of 96.2%.

### Secondary endpoint: myocardial fibrosis

**Figure 3:**
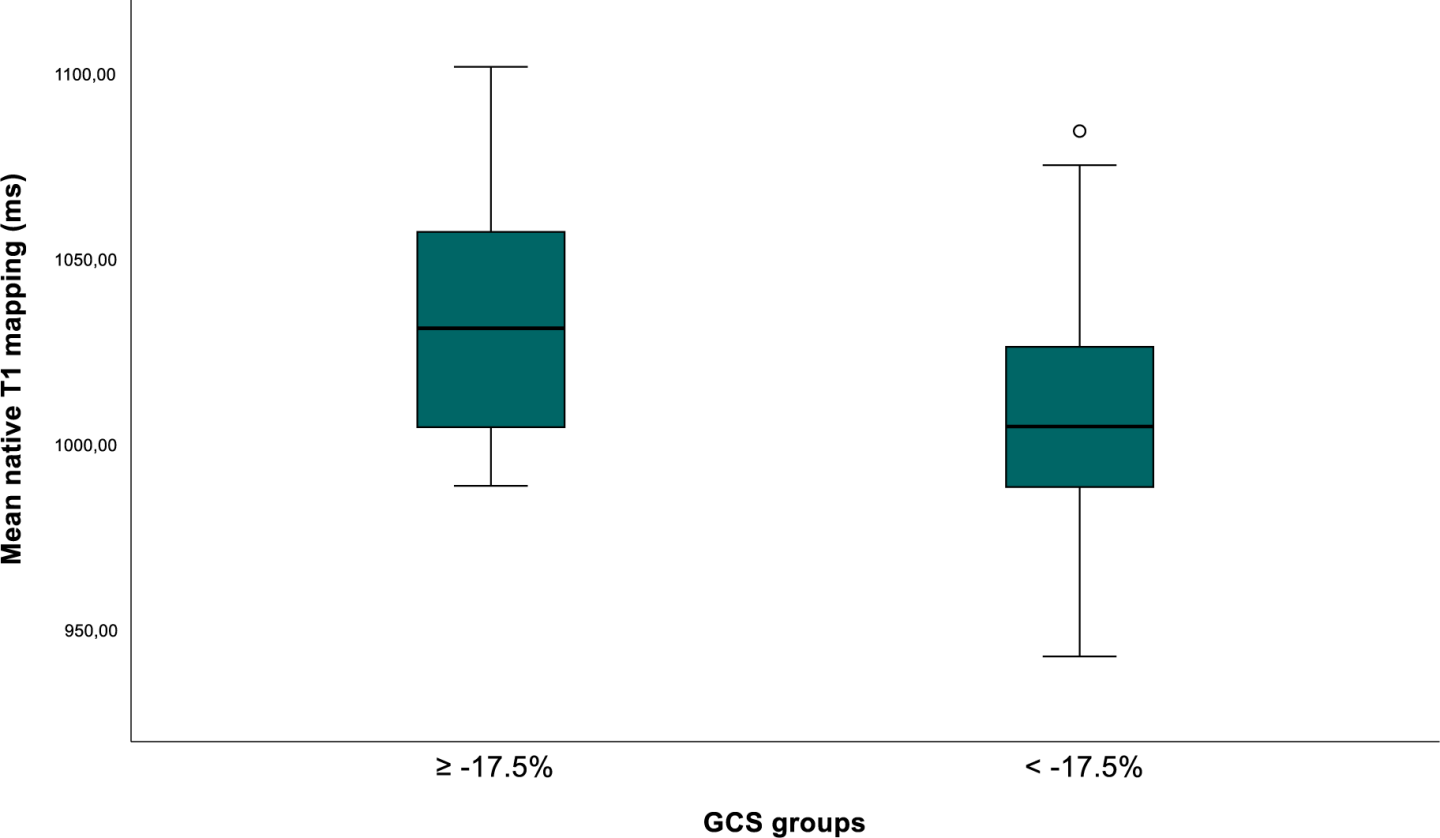
Boxplot of native myocardial T1-mapping values and GCS. Boxplot comparing native T1-mapping values between patients with GCS higher/equal and lower than −17.5%. GCS, global circumferential strain.

In the univariate analysis, we identified an association between native T1-mapping values and worse GCS (p=0.06), elevated EF (p=<0.001), but not with GLS (p=0.77). Patients with GCS≥−17.5% had significantly higher mean native T1-mapping values compared with those with GCS<−17.5% (1035 ms vs 1009 ms, p=0.03) (figure 3). Similarly, patients with EF≤46.5% exhibited higher native T1-mapping values than those with EF>46.5% (1031 ms vs 1008 ms, p=0.02).

Further univariate analysis of myocardial fibrosis revealed that increased ESV (p=<0.001) and EDV (p=0.001), percentage of aortopulmonary collaterals (p=0.01) and shunt type (right ventricular-pulmonary artery (RV-PA)) (p=0.02) were all associated with elevated native T1-mapping values (online supplemental table 2). In multivariable analysis, increased ESV (b=0.5 (95% CI 0.3 to 0.9), p=<0.001) was associated with higher native T1-mapping values (table 2).

Additionally, we further examined the relationship between the type of pre-Glenn source of pulmonary blood flow and T1-mapping values. Patients with a history of RV-PA shunt had higher mean native T1-mapping values (1027 ms, SD=31.4) compared with those with a Blalock–Taussig shunt (1001 ms, SD=31.7) or pulmonary artery banding (1006 ms, SD=25.6).

Specifically, the RV-PA group demonstrated the highest native T1-mapping values in both the lateral and septal walls. In the lateral wall, T1 values were significantly elevated (1019 ms, SD=36.3) compared with the Blalock–Taussig (989 ms, SD=24.1) and pulmonary artery banding (997 ms, SD=28.0) groups (p=0.002). Similarly, in the septal wall, the RV-PA group showed higher T1 values (1040 ms, SD=44.0) than the Blalock–Taussig (1007 ms, SD=43.0) and pulmonary artery banding (1008 ms, SD=22.0) groups (p=0.016).

**Figure 4:**
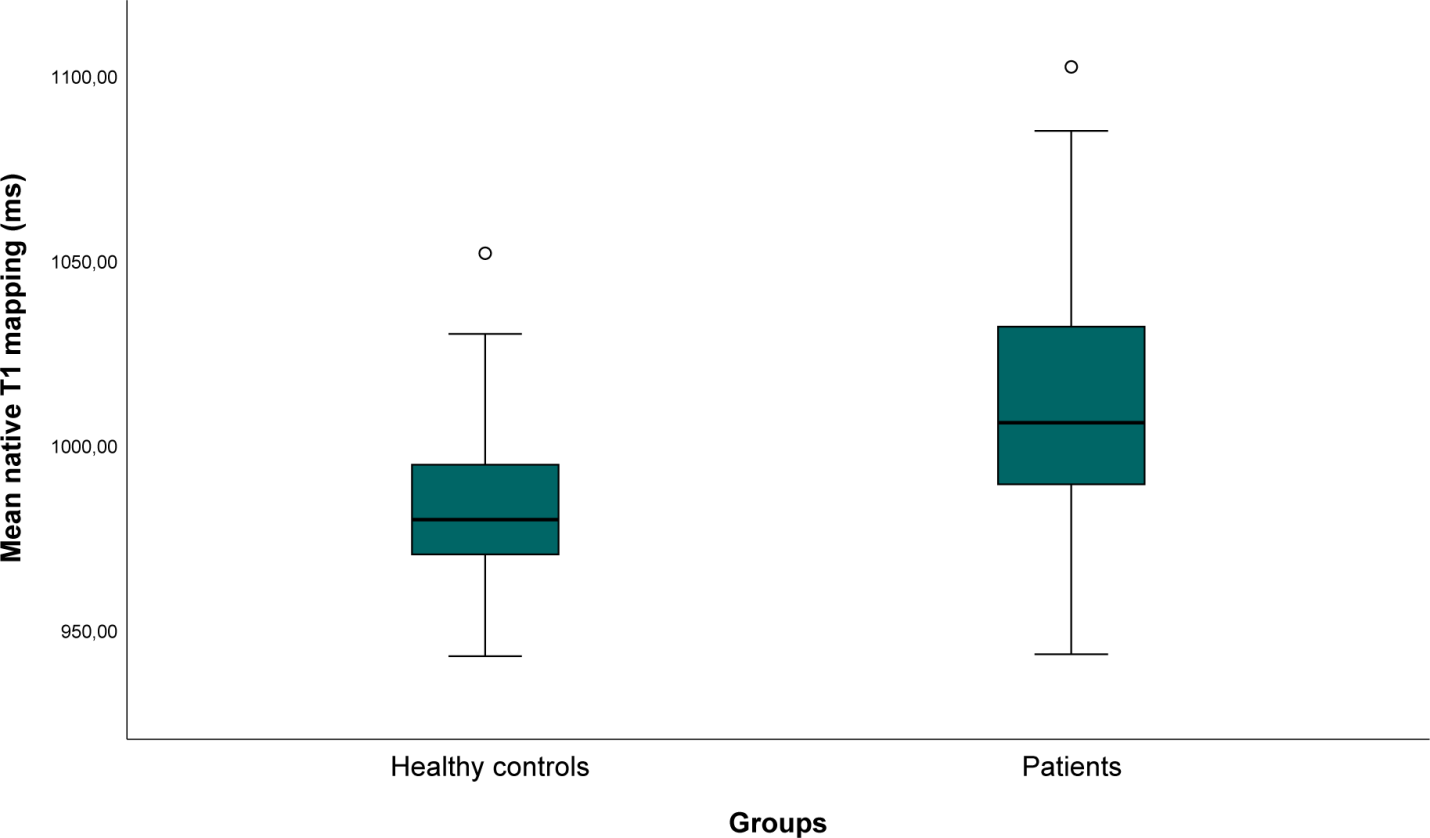
Boxplot of native T1-mapping values in patients versus healthy controls. Boxplot comparing native myocardial T1-mapping values between healthy controls and patients.

No statistically significant differences in native T1-mapping values were observed in the inferior walls among patients with RV-PA shunt, Blalock–Taussig shunt and pulmonary artery banding. Furthermore, the mean native T1-mapping values for healthy controls were significantly lower than those for patients with Fontan circulation (986 ms vs 1011 ms, p<0.001) (figure 4).

## Discussion

In this retrospective nationwide study, we assessed the clinical outcomes and their risk factors in a carefully selected cohort of 148 Fontan patients. We found that 4% of the participants met the primary endpoint during the follow-up. We observed significant correlations among poorer GLS, GCS and reduced EF with the primary endpoint. Additionally, we identified an association between worsening cardiac function and secondary endpoint of higher native T1-mapping values, suggesting myocardial fibrosis as a potential contributor to adverse outcomes.

### Primary endpoints

This study involved a highly prioritised and relatively homogeneous Fontan patient cohort, characterised by a lower baseline risk of mortality and transplantation compared with other studies.^8^ The low number of hard clinical endpoints of our patients can be attributed to our stringent national surgical criteria in this challenging patient population. There was a notable difference in the proportion of patients reaching the defined endpoint of death or transplantation, with the study by Meyer *et al.* reporting 14% (57 out of 416) compared with our 4.1% (6 out of 148). This despite similar times between Fontan surgery and CMR examination in both studies (12.6 years and 10.8 years, respectively).^6^

Further, large-scale multicentre research is needed to compare outcomes between low- and high-risk patient groups. Such studies could provide valuable insights into risk stratification and inform management strategies across the broader Fontan population.^15^

Despite the generally more favourable baseline characteristics of our patient population, our study identifies similar predictors for adverse events.^67 9 16^,^18^ The association of impaired GLS and GCS with poor outcomes is particularly significant. Our results suggest that a GLS threshold of −16.8% and a GCS threshold of −17.5% are important predictive markers, as shown by their high specificity in ROC analysis. In addition, these thresholds fall below the levels reported in healthy children.^19 20^ These findings highlight the potential of CMR-derived strain measurements for risk stratification in Fontan patients. While the sensitivity was modest, both GLS and GCS demonstrated high NPVs, suggesting that they could be useful in identifying patients at lower risk for adverse outcomes. However, further research is needed to fully establish their clinical utility.

In addition to haemodynamic risk factors, we identified late-stage clinical parameters—such as arrhythmias, PLE, NYHA functional class ≥2 and liver cirrhosis—that significantly increased the likelihood of reaching the primary endpoint. These findings align with previous research, emphasising the multifactorial nature of risk in Fontan patients.^67 9 16^,^18^

Interestingly, thoracic lymph anomalies were not found to be a statistically significant risk factor. Contrary to expectations, higher grade anomalies were more common among patients who did not experience adverse outcomes. This contrasts with PLE, a well-established risk factor, which was more common among patients with worse outcomes in our study. This unexpected finding warrants further investigation to better understand its implications.

### Myocardial fibrosis

We performed an additional analysis of myocardial fibrosis as a secondary endpoint due to its previously known significant association with adverse outcomes in Fontan patients.^9^,^1116^ Native T1 mapping was introduced later during the study period, leading to missing values. We observed a significant association between native T1-mapping values and both GCS and EF. Specifically, patients with worse GCS and EF exhibited higher native T1-mapping values, suggesting altered myocardial mechanics and increased fibrotic remodelling. This is particularly relevant, as myocardial fibrosis is increasingly recognised as a contributor to adverse outcomes in heart disease.^9^,^11^

Additionally, our findings suggest an influence of increased ESV and shunt type on native T1-mapping values, indicating that these factors may also contribute to the observed myocardial changes. Notably, patients with a history of RV-PA shunts exhibited higher native T1-mapping values compared with those with other shunt types. Larger studies are needed to confirm these findings.

### Limitations

Limitations of the study include the small number of patients reaching the endpoint. This is an important consideration when interpreting the results. Additionally, we encountered missing values for myocardial fibrosis due to the relatively recent implementation of native T1 mapping in clinical practice compared with the longer established use of CMR follow-ups. While extracellular volume may demonstrate a stronger correlation with myocardial fibrosis, its reliance on contrast agents makes T1 mapping a more practical alternative.

The strengths of our study include the use of a nationwide cohort with centralised surgical care, ensuring consistent treatment and surgical techniques across the population. All patients with Fontan circulation in our centre undergo scheduled follow-up CMR, eliminating referral bias. Furthermore, the high-quality registries in Finland facilitate comprehensive data collection for our cohort.

In conclusion, this study shows that only 4% of Fontan patients in our cross-sectional cohort either died or were listed for heart transplantation, highlighting the effectiveness of thorough cohort patient selection for the Fontan procedure. CMR imaging emerged as a convenient tool for assessing risk for adverse outcomes. In line with previous research, our findings indicate that declining myocardial function—assessed through GLS, GCS and EF—is significantly associated with adverse outcomes, reinforcing their value as predictive markers. Furthermore, the study highlights the multifactorial nature of risk, with particular emphasis on the impact of myocardial fibrosis on ventricular function.

While limitations, such as a small sample size, exist, the strengths of a centralised surgical approach bolster the reliability of our findings. Further research with larger cohorts is needed to validate these predictors and refine risk stratification strategies.

## Supplementary material

10.1136/openhrt-2025-003306 online supplemental table 1

## Data Availability

Data are available on reasonable request.
