# Supplementary material for: Cardiac MRI in patients with Fontan circulation: assessing risk factors for adverse outcomes
Source: Open Heart. 2025 May 21;12(1):e003306. doi: 10.1136/openhrt-2025-003306 (PMC12096989; doi:10.1136/openhrt-2025-003306)
Supplement: online supplemental table 1 [file openhrt-12-1-s001.docx]

**Supplemental material**

| **Table S1 Univariate analysis of GLS, GCS and EF** | | | | |  |  |  |
| --- | --- | --- | --- | --- | --- | --- | --- |
| **Categorical variables** | **GLS (N=146)** | **P-value** | **GCS  (N=142)** | **P-value** |  | **EF (N=148)** | **P-value** |
| Sex  Male Female | -21.8 [5.8] -21.6 [4.1] | 0.84 | -23.7 [5.5] -25.3 [5.8] | 0.094 |  | 55.9 [8.7] 54.7 [10.6] | 0.39 |
| Type of defect  HLHS Other UVH | -20.6 [4.5] -22.6 [5.5] | 0.02 | -23.2 [5.5] -25.4 [5.6] | 0.02 |  | 51.6 [9.2] 57.1 [9.2] | 0.27 |
| NYHA functional class $\geq2$ Yes No | -19.4 [4.7] -22.0 [5.1] | 0.12 | -19.6 [5.0] -24.7 [5.5] | 0.003 |  | 50.6 [8.8] 55.8 [9.4] | 0.28 |
| Arrhytmias Yes No | -20.8 [4.5] -21.7 [5.3] | 0.55 | -22.4 [5.1] -24.5 [5.7] | 0.22 |  | 55.9 [8.7] 55.9 [8.7] | 0.37 |
| Liver cirrhosis Yes  No | -19.9 [5.0] -21.9 [5.2] | 0.17 | -20.6 [5.7] -24.7 [5.5] | 0.01 |  | 49.6 [8.5] 55.4 [9.5] | 0.21 |
| Lymph $\geq2$ [thorax] Yes No | -21.8 [5.1] -21.6 [5.0] | 0.81 | -24.1 [5.4] -24.8 [6.3] | 0.56 |  | 54.5 [9.0] 55.5 [10.7] | 0.82 |
| Lymph [abdomen] Yes No | -22.7 [6.3] -21.3 [4.9] | 0.27 | -23.2 [6.7] -24.3 [5.5] | 0.48 |  | 54.5 [9.0] 51.6 [11.5] | 0.18 |
| Protein losing enteropathy Yes  No | -20.0 [4.6] -21.8 [5.2] | 0.45 | -22.0 [5.3] -24.4 [5.7] | 0.34 |  | 51.0 [9.6] 55.0 [9.5] | 0.42 |
| AVVR  Yes No | -21.0 [4.9] -23.7 [5.6] | 0.04 | -23.8 [5.7] -25.8 [5.1] | 0.18 |  | 54.3 [9.8] 56.5 [8.6] | 0.11 |
| Shunt type  RV-PA  BT  PA-banding | -20.8 [4.2] -22.6 [5.1] -21.7 [5.2] | 0.15 | -22.7 [5.4] -25.3 [6.1] -24.6 [4.6] | 0.099 |  | 51.4 [10.0] 56.4 [8.8] 58.4 [8.0] | 0.04 |
| **Continuous variables** | **Pearson correlation coefficient, GLS** | **P-value** | **Pearson correlation coefficient, GCS** | **P-value** |  | **Pearson correlation coefficient, EF** | **P-value** |
| Age at Fontan [years] | 0.02 | 0.85 | 0.08 | 0.35 |  | -0.07 | 0.33 |
| Age at CMR [years] | 0.03 | 0.72 | 0.07 | 0.38 |  | 0.03 | 0.18 |
| Time from Fontan to CMR [years] | 0.02 | 0.83 | 0.05 | 0.59 |  | 0.04 | 0.13 |
| GLS [%] | - | - | 0.34 | <0.001 |  | -0.25 | 0.002 |
| GCS [%] | 0.34 | <0.001 | - | - |  | -0.52 | <0.001 |
| EF [%] | -0.26 | 0.002 | -0.58 | <0.001 |  | - | - |
| Saturation at CMR [%] | 0.07 | 0.45 | 0.12 | 0.17 |  | 0.12 | 0.12 |
| T1 mapping value [ms] | -0.03 | 0.77 | 0.21 | 0.06 |  | -0.37 | <0.001 |
| CI | -0.03 | 0.71 | -0.14 | 0.11 |  | 0.20 | 0.04 |
| EDV [ml/m²] | 0.13 | 0.13 | 0.36 | <0.001 |  | -0.45 | <0.001 |
| ESV [ml/m²] | 0.12 | 0.15 | 0.45 | <0.001 |  | -0.79 | <0.001 |
| Pulmonary perfusionratio | 0.07 | 0.43 | -0.04 | 0.68 |  | 0.01 | 0.79 |
| Aortopulmonary collaterals [%] | 0.07 | 0.39 | 0.09 | 0.27 |  | -0.11 | 0.02 |
| Values are reported as N (%), median (25^th^ ,75^th^ percentile) or mean [standard deviation]. HLHS = hypoplastic left heart syndrome, NYHA = New York Heart Association, PLE = protein-losing enteropathy, CMR = cardiac magnetic resonance, RV-PA = right ventricle – pulmonary artery, BT = Blalock Taussig, PA = pulmonary artery, AVVR = atrioventricular valve regurgitation, GLS = global longitudinal strain, GCS = global circumferential strain, EF = ejection fraction, CI = cardiac index, EDV = end-diastolic volume, ESV = end-systolic volume | | | | |  |  |  |

| Table S2. Univariable analysis T1 mapping (N=83) | | |
| --- | --- | --- |
| **Variable** | **T1** | **P-value** |
| Sex  Male Female | 1011 [34.3] 1012 [28.5] | 0.81 |
| Type of defect  HLHS Other UVH | 1012 [32.8] 1011 [31.1] | 0.92 |
| NYHA functional class $\geq2$ Yes No | 1003 [34.4] 1012 [31.7] | 0.61 |
| Arrhythmias Yes No | 1022 [29.6] 1008 [32.0] | 0.11 |
| Liver cirrhosis Yes  No | 1021 [34.4] 1011 [31.4] | 0.37 |
| Lymph $\geq2$ [thorax] Yes No | 1009 [30.2] 1020 [37.6] | 0.24 |
| Lymph [abdomen] Yes No | 1017 [43.1] 1011 [29.7] | 0.56 |
| Protein losing enteropathy Yes  No | 1023 [22.4] 1011 [31.9] | 0.58 |
| AVVR  Yes No | 1013 [32.3] 1011 [30.8] | 0.77 |
| Shunt type  RV-PA  BT  PA-banding | 1027 [31.4] 1001 [31.7] 1006 [25.6] | 0.016 |
| Age at Fontan [years] | 0.08 | 0.47 |
| Age at CMR [years] | -0.23 | 0.04 |
| Time from Fontan to CMR [years] | -0.23 | 0.04 |
| GLS [%] | -0.03 | 0.77 |
| GCS [%] | 0.21 | 0.06 |
| EF [%] | -0.37 | <0.001 |
| Saturation at CMR [%] | -0.18 | 0.11 |
| CI | 0.04 | 0.72 |
| EDV [ml/m²] | 0.35 | 0.001 |
| ESV [ml/m²] | 0.47 | <0.001 |
| Pulmonary perfusionratio | -0.02 | 0.83 |
| Aortopulmonary collaterals [%] | 0.28 | 0.01 |
| Values are reported as N (%), median (25^th^ ,75^th^ percentile) or mean [standard deviation]. HLHS = hypoplastic left heart syndrome, NYHA = New York Heart Association, PLE = protein-losing enteropathy, CMR = cardiac magnetic resonance, RV-PA = right ventricle – pulmonary artery, BT = Blalock Taussig, PA = pulmonary artery, AVVR = atrioventricular valve regurgitation, GLS = global longitudinal strain, GCS = global circumferential strain, EF = ejection fraction, CI = cardiac index, EDV = end-diastolic volume, ESV = end-systolic volume | | |
